# Supplementary material for: Impact of the 1918 Influenza Pandemic in Coastal Kenya
Source: Trop Med Infect Dis. 2019 Jun 8;4(2):91. doi: 10.3390/tropicalmed4020091 (PMC6631354; doi:10.3390/tropicalmed4020091)
Supplement: Supplementary file 1 [file tropicalmed-04-00091-s001.pdf]

Table S1. Numbers and rates of overall health care use and all-cause mortality by year in Coast Province Kenya, 1912-1925

	Calendar Year													
	1912	***1913***	***1914***	***1915***	***1916***	***1917***	***1918***	***1919***	***1920***	***1921***	***1922***	***1923***	***1924***	***1925***
Population Estimate	203033	234982	243775	243841	223725	180537	170578	181283	204572	197088	200267	170000	208505	202516
Number of Districts with information	7	7	7	7	7	5	5	5	7	5	7	7	7	7
NumberIn-Patient & Out-Patients Cases	310*	6454	6152	7957	6705	410*	25047	23832	19036	18535	15656	19032	25974	17996
Number of Districts with information	1	7	7	7	7	1	5	5	7	5	7	7	7	7
Number of All-Cause Deaths	96	226	757	688	712	722	4166	1476	2880	*2631*	*3345*	*2822*	*2732*	*3949*
Number of Districts with information	7	7	7	7	7	5	5	5	7	5	7	7	7	7
Crude All-cause Mortality Rates¥	0.5	1.0	3.1	2.8	3.2	4.0	24.4	8.1	14.1	13.3	16.7	16.6	13.1	19.5
Crude inpatient and outpatient Rates¥	8.6	27.5	25.2	32.6	30.0	11.1	146.8	131.5	93.1	94.0	78.2	112.0	124.6	88.9

**Missing data, only Mombasa Island data reported ¥ rates (/1000 people/year) Note: Years 1917, 1918, 1919 and 1921 data for Lamu and Tana River Districts missing*

List S1. List of References from Kenya National Archive Library, Nairobi

Population denominators, 1912-1925

British-Colonial-Office. 1917b. “Census Records (1917).” KNA/PC/COAST/1/1/361.
———. 1918a. “Blue Book (1917-1918).” KNA/PC/COAST/1/20/40.
———. 1918b. “Population Estimates (1915-1918).” KNA/PC/COAST/1/1/328.
———. 1920. “Official Directory of Population(1915-1920).” KNA/PC/COAST/1/1/407.
———. 1923e. “Estimates of Population (1918-1923).” KNA/PC/COAST/1/1/267.
———. 1926b. “Coastal Zone Administration (1915-1926).” KNA/PC/COAST/1/1/405.
———. 1927a. “District Gazzeter (1917-27).” KNA/PC/COAST/1/20.
———. 1927c. “Registration of Births and Deaths (1916-1928).” KNA/PC/COAST/1/1/230.
———. 1928a. “Administration of Native Reserves (1915-1928).” KNA/PC/COAST/1/1/133.
———. 1912. British-East-Africa-Protectorate. “Hut Counting (1912).” KNA/PC/COAST/1/3/67.

Health facility use and all-cause mortality, 1912-1925

British-Colonial-Office. 1912. Annual East Africa Protectorate (Kenya) Annual Report for 1912-1913.
———. 1912. British-East-Africa-Protectorate. “Hut Counting (1912).” KNA/PC/COAST/1/3/67.
———. 1914. “East Africa Protectorate Report For 1914-15.” Colonial Reports—Annual (881): 1–42.
———. 1915a. “East Africa Protectorate. Report For 1915-16.” Colonial Reports—Annual (921): 1–24.
———. 1915b. “East Africa Protectorate Report For 1913-14.” Colonial Reports-Annual (840): 1–77.
———. 1916. “East Africa Protectorate. Report For 1916-17.” Colonial Reports-Annual (988): 1–26.
———. 1917. “East Africa Protectorate. Report For 1917-18.” Colonial Reports-Annual (1013): 1–28.
———. 1918. “East Africa Protectorate. Report For 1918-19.” Colonial Reports—Annual (1073): 1–26.
———. 1919. “East Africa Protectorate. Report For 1919-20.” Colonial Reports-Annual (1089): 1–28.
———. 1920. “Report On The Colony And Protectorate Of Kenya For The Year 1920.” Colonial Reports-Annual 194(1188):
———. 1921. “Report On The Colony And Protectorate Of Kenya For The Year 1921.” Colonial Reports—Annual (1153):
———. 1922. “Report On The Colony And Protectorate Of Kenya For The Year 1922.” Colonial Reports—Annual (1188):
———. 1923. “Report On The Colony And Protectorate Of Kenya For The Year 1923.” Colonial Reports—Annual (1227):
———. 1924. “Report On The Colony And Protectorate Of Kenya For The Year 1924.” Colonial Reports—Annual (1282):
———. 1925. “Report On The Colony And Protectorate Of Kenya For The Year 1925.” Colonial Reports—Annual (1321):
———. 1926. “Report On The Colony And Protectorate Of Kenya For The Year 1926.” Colonial Reports—Annual (1352).
———. 1927. “Report On The Colony And Protectorate Of Kenya For The Year 1927.” Colonial Reports—Annual (1425):
———. 1928. “Medical Department Annual Reports(1916-1928).” KNA/PC/COAST/1/15/115.
———. 1914a. “Annual Medical Report(1912-1914).” KNA/PC/COAST/1/15/45.

———. 1917a. “Annual Report-Seyidie Province(1917).” KNA/PC/COAST/1/12/260 6(3)
———. 1922a. “Annual Report(1910-1922)-Malindi.” KNA/DC/MAL/1/1.
———. 1922b. “Annual Report(1922).” KNA/AVW/11/52.
———. 1923b. “Annual Report, Seyidie (1920-1923)”. KNA/PC/Coast/1/20/112.
———. 1923c. “Annual Report(1913-1923)-Nyika.” KNA/DC/KFI/1/1.
———. 1923d. “Annual Report(1920-1923)-Seyidie.” KNA/PC/COAST/1/20/112.
———. 1924. “Coast Province Annual Report(1924).” KNA/PC/COAST/1/2/33.
———. 1925a. “Annual Report(1925)-.” KNA/AVW/11/53.
———. 1925b. “Coast Province Annual Report(1925).” KNA/PC/COAST/2/1/32.
———. 1926a. “Coast Province Annual Reports (1926).” KNA/PC/COAST/2/1/31.
———. 1928b. “Annual Report(1921-1928)-Lamu.” KNA/DC/LAM/1/1.
———. 1928c. “Annual Report(1923-1928)-Malindi.” KNA/DC/MAL/1/2.
———. 1928d. “Sanitation and Other Medical Reports(1925-1928).” KNA/PC/COAST/1/15/119.
———. 1930. “Annual Report(1921-1930)-Mombasa.” KNA/DC/MSA/1/3.
———. 1931. “Annual Report(1924-1931)-Nyika.” KNA/DC/KFI/1/2.
———. 1937. “Annual Report(1923-1937)-Taita.” KNA/DC/TTA/1/1.

Pandemic Influenza, 1918-1919

British-Colonial-Office. 1919. “Spanish Influenza (1918-1919).” KNA/PC/COAST/1/1/369.
———. 1923a. “Annual District Reports(1919-1923).” KNA/PC/COAST/1/1/412.
———. 1927b. “Medical Attendance in Native Reserves(1918-27).” KNA/PC/COAST/1/15/117.
———. 1923b. “Annual Report, Seyidie (1920-1923)”. KNA/PC/Coast/1/20/112.
———. 1923c. “Annual Report(1913-1923)-Nyika.” KNA/DC/KFI/1/1.
———. 1923d. “Annual Report(1920-1923)-Seyidie.” KNA/PC/COAST/1/20/112.
———. 1918. “East Africa Protectorate. Report For 1918-19.” Colonial Reports—Annual (1073): 1–26.
———. 1919. “East Africa Protectorate. Report For 1919-20.” Colonial Reports-Annual (1089): 1–28.
———. 1920. “Report On The Colony And Protectorate Of Kenya For The Year 1920.” Colonial Reports-Annual 194(1188):
———. 1928. “Medical Department Annual Reports(1916-1928).” KNA/PC/COAST/1/15/115.
———. 1919. "Annual Reports from Districts 1918/19", Vanga Annual, p.1,4; KNA/PC/COAST/1/1/266
———. 1919. "Porters for Military". KNA/PC/COAST/1/13/118
East African Standard 10.9.1918. Provides information on the introduction of pandemic in Kenya
———. 1919. "Annual Reports from Districts 1918/19", Malindi district KNA/PC/COAST/1/1/266 and
———. Malindi Report, p.7; KNA/HEALTH/1/10037
